# COVID-19 associated multisystem inflammatory syndrome in children mimicking acute appendicitis - how to differentiate and conduct pediatric patients during the pandemic? - Proposal of a management flowchart

**DOI:** 10.1590/0100-6991e-20213012

**Published:** 2021-12-17

**Authors:** LORAINE ENTRINGER FALQUETO, CAROLINA MARTINS VISSOCI, ISABELLA CRISTINA BONETTO FERREIRA, AMANDA GINANI ANTUNES, FERNANDO ANTÔNIO BERSANI AMADO, SYLVIO ANDRADE GILBERTO AVILLA, CLAUDIO SCHULZ, FABIO ARAUJO MOTTA, ELISANGELA DE MATTOS E SILVA

**Affiliations:** 1 - Hospital Pequeno Principe, Departamento de Cirurgia Pediatrica - Curitiba - PR - Brasil; 2 - Hospital Pequeno Principe, Departamento de Infectologia, Núcleo de Pesquisa Clínica e Núcleo da Qualidade - Curitiba - PR - Brasil

**Keywords:** Systemic Inflammatory Response Syndrome, COVID-19, Pediatrics, Acute Abdomen, Appendicitis, Síndrome de Resposta Inflamatória Sistêmica, COVID-19, Pediatria, Abdome Agudo, Apendicite

## Abstract

**Introduction:**

the new coronavirus pandemic has been a reality throughout 2020, and it has brought great challenges. The virus predominantly manifests in the pediatric population with mild symptoms. However, an increase in the incidence of Multisystemic Inflammatory Syndrome in Children (MIS-C) associated with COVID-19 has been described in the literature. MIS-C manifests mainly with fever and gastrointestinal symptoms and may mimic acute abdomen due to acute appendicitis. The objective of this study is to propose a care flowchart for suspected cases of acute appendicitis in the initial phase in pandemic times, considering the possibility of MIS-C. This situation was brought up by a patient treated in a pediatric hospital in Brazil.

**Discussion:**

It was possible to identify common signs and symptoms in the reported patient and those published cases that may serve as alerts for early identification of MIS-C cases. Based on the literature review and on the similarities between the syndrome and the inflammatory acute abdomen in children, we elaborated an initial approach for these cases to facilitate the identification, early diagnosis, and management. The flowchart considers details of the clinical history, physical examination, and complementary exams prior to the indication of appendectomy in patients with initial phase symptoms.

**Conclusion:**

MIS-C, although rare and of poorly known pathophysiology, is most often severe and has a high mortality risk. The use of the proposed flowchart can help in the diagnosis and early treatment of MIS-C.

## RESUMO

**Introdução:**

a pandemia do novo coronavírus arrastou-se ao longo de 2020 e trouxe grandes desafios. Acredita-se que o vírus manifesta-se na população pediátrica predominantemente com quadros leves, entretanto, aumento da incidência da Síndrome Inflamatória Multissistêmica em Crianças (SIM-C) associada à COVID-19 tem sido descrito na literatura. A SIM-C manifesta-se principalmente com febre e sintomas gastrointestinais, podendo mimetizar abdome agudo inflamatório por apendicite aguda. O objetivo deste trabalho é propor fluxograma de atendimento dos casos suspeitos de apendicite aguda em fase inicial, em tempos de pandemia, considerando-se a possibilidade de SIM-C, motivado pelo caso de paciente atendido em hospital pediátrico no Brasil.

**Discussão:**

Foi possível identificar sinais e sintomas em comum entre o paciente aqui relatado e casos publicados que podem servir de alerta para identificação precoce dos casos de SIM-C. Com base na revisão da literatura e nas semelhanças entre a síndrome e quadros de abdome agudo inflamatório na criança, foi elaborado fluxograma de abordagem inicial destes doentes para facilitar a identificação, diagnóstico precoce e condução dos pacientes. O fluxograma leva em consideração detalhes da história clínica, exame físico e exames complementares antes da indicação de apendicectomia em pacientes com sintomas na fase inicial.

**Conclusão:**

A SIM-C, apesar de rara e da fisiopatologia pouco conhecida, apresenta-se na maioria das vezes de forma grave e possui alto risco de mortalidade. O uso do fluxograma proposto pode auxiliar no diagnóstico e tratamento precoce da SIM-C.

## INTRODUCTION

The pandemic of the new coronavirus has been a reality throughout 2020 and brought many challenges with it. It is believed that the virus manifests in the pediatric population predominantly with milder clinical pictures. However, in April of 2020 an increase in the incidence of Kawasaki like Syndrome and hyperinflammatory shock was reported in England, with potential association to COVID-19^1^ ^,^ ^2^. Since then, the same has been described in other countries as Multisystem Inflammatory Syndrome in Children (MIS-C) or Pediatric Multisystem Inflammatory Syndrome (PMIS), which became part of the COVID-19 clinical manifestations spectrum^3^ ^-^ ^10^.

MIS-C includes a hyperimmune inflammatory phase, whose main signs and symptoms are fever and gastrointestinal manifestations and may simulate acute abdomen due to acute appendicitis in children^11^. 

Considering the high incidence of acute appendicitis as cause of acute abdomen in pediatric emergency services worldwide, to rethink the investigation flow of these patients in the current context of the SARS-COV-2 pandemic becomes fundamental to avoid unnecessary operations in children affected by MIS-C. As far as we know, even in face of such a challenge, no publication has proposed a systematic approach to guide the diagnosis of these patients in reference care providers. 

The flowchart to assist in the diagnosis of MIS-C cases is even more important in lieu of the increase in cases since early 2021. This inflammatory syndrome tends to be present in children in approximately four weeks after the infection, with or without signs or symptoms. 

The objective of this work is to propose a flowchart of care to patients with suspected acute abdomen in pandemic times, considering the possibility of MIS-C. It is important to point out that the drafting of the flowchart had as great motivator one case of MIS-C diagnosed in the immediate appendectomy postoperative period, herein reported. The initial approach flowchart aims to facilitate identification, early diagnosis, and suitable treatment for patients with possible MIS-C associated with, or simulating, acute appendicitis.

## METHOD

The motivation for this study is the case of a patient attended at the Hospital Pequeno Príncipe, Curitiba, Parana State, Brazil, with diagnosis of COVID-19 associated Multisystem Inflammatory Syndrome, who had initial diagnosis and treatment of acute appendicitis, and is herein reported. 

The proposal of a flowchart was presented and discussed with an interdisciplinary team composed of members of the services of Pediatric Surgery, Pediatrics, Infectious Diseases, Cardiology, and Intensive Therapy. To support the construction of this flowchart, we reviewed the major published studies through a search in Pubmed from June to December 2020, to identify the relationship of such cases with the clinical picture of suspected acute appendicitis. 

This work was analyzed and approved by the Ethics in Research Committee of the Hospital Pequeno Príncipe (opinion number 4464260). 

### Case report

A male, 12-year-old, previously healthy patient, with body mass index (BMI) percentile in the 50-85 range, admitted to the ER with abdominal pain starting three days before associated with fever higher than 38.5°C. He evolved with vomiting and diarrhea on the third day. He did not have any respiratory complaints, nor reported having had contact with people who got sick in the near past. At physical exam, he displayed tachycardia, with cold extremities and pain at abdominal palpation, mainly in the right iliac fossa, with signs of diffuse peritoneal irritation. Initial complementary exams showed leukocyte count of 7,310/µL with 24% of rods, 143,000/mm³ platelets, extended prothrombin activity, with INR 1.6, and C reactive protein (CRP) of 79.5mg/L. 

Due to the clinical picture and the physical examination highly suggestive of acute appendicitis, with Alvarado score of 812, the patient underwent conventional appendectomy, without the need of further imaging studies. Despite the lingering clinical picture, the intraoperatively finding was of early, uncomplicated acute appendicitis, with blockade in right iliac fossa and a large amount of hematic fluid in the abdominal cavity. The pathology of the specimen showed acute focal periapendicitis with lymphoid hyperplasia and vascular congestion. 

On the third postoperative day (PO), the patient presented with dyspnea, pain in the cervico-thoracic spine region, anasarca, oliguria, decreased oxygen peripheral saturation, hypotension, and fever persistence. He was transferred to the intensive care unit (ICU) for monitoring due to clinical worsening, but remained hemodynamically stable, without the need for vasoactive drugs. In view of the atypical evolution, new exams were collected. CRP was 246mg/L; tests for COVID-19: negative RT-PCR, negative IgM, and positive IgG. In the fifth PO, he presented with urticarial plaques in the skin of the inferior limbs, mental confusion, involuntary movements, psychomotor agitation, and erythema of the eyelids, ears, and inguinocrural region. During all the postoperative period, he had bilious vomiting and mucous diarrhea, without blood. 

Ten days after the beginning of symptoms, the patient became hypertensive and displayed sinus bradycardia, with widened QT segment. Transthoracic ultrasound revealed an ejection fraction (EF) of 70% and increase in the diameters of the left and right coronary arteries, 3.5mm and 4.0mm, respectively. In face of the diagnosis of COVID-19 associated MIS-C, treatment was started with acetylsalicylic acid 100 mg/kg/day and immunoglobulin 2g/kg/day, for one day. He evolved with improvement of signs and symptoms and cardiac changes, being discharged after 20 days of admission, twelve of them in the ICU. 

After discharge, the patient did not present new complications related to the reported condition, completing 12 months of outpatient follow-up with the Pediatrics, Pediatric Surgery, and Pediatric Cardiology.

## DISCUSSION

The manifestation of COVID-19 in children is spectral, from asymptomatic to the most serious ones. There is not yet a specific definition of the actual prevalence of each presentation, how often is the progression, and how the worsening the condition may become. Factors determining the worst outcomes have not been defined. 

Despite the little knowledge about the immune response to COVID-19, it is believed that MIS-C is associated with the development of acquired immunity about four weeks after the primary infection, with greater prevalence in otherwise healthy school children and adolescents^10^ ^,^ ^13^ ^-^ ^17^. The definition of MIS-C includes clinical and laboratory features, with evidence of COVID-19 infection or contact with people who have or had the disease^5^ ^,^ ^18^. A limitation to this confirmation is the difficulty of access to the disease’s serological and confirmatory tests in all cases. 

Pediatric patients with fever, abdominal pain located in right iliac cavity, and vomiting point to acute inflammatory abdomen, whose main cause is acute appendicitis, for which the diagnosis is eminently clinical, as the case presented here. For such cases there is no obligation of further imaging studies. However, since MIS-C signs and symptoms can be very similar to the ones of initial acute appendicitis, it becomes essential to distinguish these conditions to avoid unnecessary surgical procedures in children with greater probability of quickly evolving to severe MIS-C. In a pandemic scenario, the diagnosis of initial acute appendicitis became a challenge and requires the use of further tests, including imaging ones. This fact changes the approach based on an exclusively clinical assessment, accepted until then. 

The specific features of the history and clinical evolution that invite the possibility of MIS-C and guide the new decision flowchart are^10^:

- Clinical history of infection by COVID-19 of the patient and/or family members in the prior three to six weeks;
- Persistent, high fever;
- Significantly high inflammatory markers;
- Physical examination incompatible with complaints;
- Imaging findings incompatible with the clinical picture.

The cases of acute inflammatory abdomen with the elements above should be evaluated with greater attention, considering PCR and serology exams for COVID-19, dosage of inflammatory markers, cardiac function markers, and imaging tests, such as sonography (considered the initial exam of choice for most cases in children), even tomography in selected cases, before diagnostic confirmation and surgical indication^10^ ^,^ ^19^. 

For the diagnosis of MIS-C, high clinical suspicion is required. Considering the immune character with a poorly defined pathophysiology and rapidly multisystemic evolution, the treatment requires monitoring in the ICU, with serial clinical and laboratory reassessments. There has been currently proposed a treatment with broad spectrum antibiotics according to the institution microbiota, medications to ventilatory and hemodynamic support, including vasoactive drugs, and, in selected cases, corticosteroids, based on international protocols^19^. When one detects cardiac changes and criteria for Kawasaki syndrome, human immunoglobulin and antiplatelet aggregation agents are used^1^ ^,^ ^7^ ^,^ ^8^ ^,^ ^10^ ^,^ ^13^ ^,^ ^16^ ^,^ ^20^. The management of the reported case was based on these literature recommendations. However, it is not possible to determine the long term evolution. 

Typically, MIS-C is characterized by high and persistent fever for three to five days, commonly associated with gastrointestinal signs and symptoms - abdominal pain, vomiting, and diarrhea^10^. Abdominal pain located in the right iliac cavity can mimic acute appendicitis, probably by lymph node involvement^3^ ^,^ ^16^ ^,^ ^17^ ^,^ ^21^ ^,^ ^22^. Skin erythema, conjunctivitis, and mucosal involvement alert to the possibility of a Kawasaki Syndrome-like condition. Neurological symptoms and signs, such as headache, confusion, and irritability, are also noted. The respiratory ones, on their turn, appear as results of severe shock, not being the most important^10^ ^,^ ^22^. Evolution to shock is present in 32% to 76% of the cases^1^ ^,^ ^3^ ^,^ ^7^ ^,^ ^9^ ^,^ ^10^ ^,^ ^13^ ^,^ ^16^ ^,^ ^17^ ^,^ ^20^ ^,^ ^23^. In severe cases, kidney and heart involvement is frequent. In the reported case, the clinical picture and the evolution were similar. 

Laboratory changes include elevation of inflammatory markers, such as CRP, D dimer, ferritin, procalcitonin, and interleukin^6^, which correspond to the progressive worsening of the condition^8^. The increase of troponin, BNP (brain natriuretic peptide) and pro-BNP levels was observed in patients with evolution to shock, alerting to the possibility of these being early markers of systemic deterioration^1^ ^,^ ^7^ ^,^ ^10^ ^,^ ^16^ ^,^ ^20^ ^,^ ^21^ ^,^ ^24^ ^,^ ^25^. The predominant echocardiographic changes are left ventricular hypofunction and coronary dilatation^16^. With installed shock, imaging exams have shown pulmonary consolidation and atelectasis, pleural effusion, free abdominal fluid, swelling of the intestinal wall, mesenteric lymphadenitis, and peri-vesicular edema^13^ ^,^ ^14^ ^,^ ^16^ ^,^ ^21^. These signs and symptoms might mimic acute appendicitis. 

The United States Centers for Disease Control and Prevention (CDC) established the diagnostic criteria for COVID-19 associated MIS-C, which include age less than 21 years with fever for more than 24 hours, laboratory evidence of inflammation, severe clinical picture requiring hospitalization, involvement of more than two organic systems (cardiac, renal, respiratory, gastrointestinal, hematological, dermatological, and neurological), absence of other plausible diagnoses, and positive infection by COVID-19 (RT-PCR, serological antigen test, or exposure to COVID-19) in the four weeks prior to the onset of signs and symptoms^8^. 

The criteria for diagnosis by WHO is a little different from those of the CDC. In short, age less than 19 years, presence of persistent fever, high inflammatory markers, and involvement of at least two systems^8^. It is important to emphasize that MIS-C is an exclusion diagnosis, and one should investigate and rule out other plausible diagnoses or obvious infectious foci that justify the condition. 

Webb K et al. reported the first 23 cases of MIS-C in patients younger than 15 years in South Africa, two of them having undergone laparotomy for suspected appendicitis. Not all cases had COVID-19 infection confirmation by antibodies research^18^. 

Interestingly, in the case herein reported, in addition to the MIS-C diagnosis, the patient also had the confirmation of acute appendicitis at histopathology. Recent publications have demonstrated that even in cases of confirmed MIS-C, the patient may also have a diagnosis of associated acute appendicitis. In a recent publication from South Africa by Vos et al., three of the four patients in the series had surgically confirmed appendicitis^11^. 

It is not clear whether appendicitis may occur as a complication of SARS-COV-2 due to lumen obstruction secondary to inflammation associated with the viral input or to reactive lymphoid hyperplasia caused by MIS-C. The major confounding factor in this scenario is the occurrence of terminal ileitis, as shown by Tullie et al. in an eight case series in London, in which no patient required surgical intervention^25^. 

Acute appendicitis is associated with Kawasaki disease, with which MIS-C shares many clinical and pathological features, possibly related to vasculitis of the appendicular artery^26^. No fecalith was found in any of the children who underwent appendectomy on the account of Vos et al., sustaining inflammation or vasculitis as pathological mechanisms^11^. 

**Figure 1:**
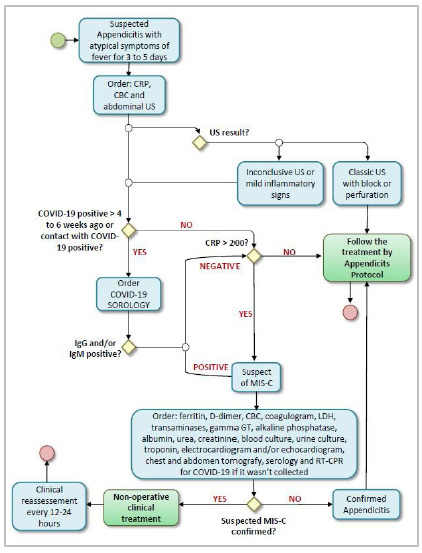
Flowchart proposed for cases of suspected appendicitis.

Motivated by the severity of MIS-C cases related to COVID-19 infection, the Pediatric Surgery Service of the Hospital Pequeno Príncipe elaborated a flowchart of care for cases of acute abdomen in children. The flowchart (Figure 1) was based on the literature review presented above and on the reported case. It applies to pediatric cases of early, acute inflammatory abdomen admitted to reference centers for the care of COVID-19 and its complications. 

Based on the available data, two possibilities must be considered in the evaluation of patients with abdominal pain suggesting early, acute abdomen in the COVID-19 pandemic scenario with suspected MIS-C: 1) the inflammatory syndrome can manifest with abdominal pain due to terminal ileitis, mimicking acute appendicitis; or 2) the inflammation process of the terminal ileum and cecal appendix can develop with obstruction of the appendicular lumen and cause acute appendicitis secondary to MIS C. 

Both the possibilities warrant high suspicion for the diagnosis of MIS-C. Due to the severity of signs and symptoms, for suspected MIS-C one should consider an initial conservative and nonoperative treatment of early and uncomplicated cases of acute appendicitis, as many protocols already adopted worldwide regardless of the pandemic context^27^. 

There is also the possibility that the surgical stress and trauma trigger or exacerbate MIS-C pictures after COVID-19 infection. There is a need for more data and follow-up of case series to confirm this hypothesis.

## CONCLUSION

From the authors experience and the literature review, it was possible to identify common features between the attended patient and other reports that can serve to alert for identification of MIS-C cases, allowing the development of the flowchart. Despite its rarity, its poorly known pathophysiology, and its difficult treatment, MIS-C is associated to severe complications and high mortality, requiring high index of suspicion for the diagnosis, reinforcing the importance of the discussion of the issue and determination of care guidelines.
